# A Novel Machine Vision System for the Inspection of Micro-Spray Nozzle

**DOI:** 10.3390/s150715326

**Published:** 2015-06-29

**Authors:** Kuo-Yi Huang, Yu-Ting Ye

**Affiliations:** 1 Department of Bio-Industrial Mechatronics Engineering, National Chung Hsing University, Tai-Chung 402, Taiwan; 2 Department of Mechatronic Engineering, Huafan University, New Taipei City 223, Taiwan; E-Mail: alexyeh1120@hotmail.com

**Keywords:** micro-spray nozzle, image processing, neural network

## Abstract

In this study, we present an application of neural network and image processing techniques for detecting the defects of an internal micro-spray nozzle. The defect regions were segmented by Canny edge detection, a randomized algorithm for detecting circles and a circle inspection (CI) algorithm. The gray level co-occurrence matrix (GLCM) was further used to evaluate the texture features of the segmented region. These texture features (contrast, entropy, energy), color features (mean and variance of gray level) and geometric features (distance variance, mean diameter and diameter ratio) were used in the classification procedures. A back-propagation neural network classifier was employed to detect the defects of micro-spray nozzles. The methodology presented herein effectively works for detecting micro-spray nozzle defects to an accuracy of 90.71%.

## 1. Introduction

Nozzles are a common mechanical component and are widely used in agriculture, manufacturing and the service industry. A micro-spray nozzle is an important component in an agricultural sprayer, which is used to spray water, pesticides and nutrients. Real-time flow uniformity depends on the internal quality of the micro-spray nozzle. Unstable flow will be created because of internal defects. The price and quality of micro-spray nozzles are down due to such defects. The internal defects of micro-spray nozzles usually result during cutting procedures with a CNC (computer numerical control) machine. Chang and Yu [1] proposed a triangular-pitch shell-and-tube spray evaporator featuring an interior spray technique. The dry-out phenomenon is prevented, and the heat transfer performance is improved in their study. In addition, micro-spray nozzles have to be sorted manually, because the surface of the micro-spray nozzle is heterogeneous, which makes auto-detection very difficult.

Image processing techniques are a powerful method and are widely used to inspect industrial products. Chen *et al.* [2] designed an automatic damage detection system of an engineering ceramic surface with image processing techniques, pattern recognition and machine vision. Paniagua *et al.* [3] proposed a neuro-fuzzy classification system to inspect the quality of industrial cork samples by incorporating techniques based on Gabor filtering techniques and neuro-fuzzy computing with three wavelet-based texture quality features. A microscopic vision system [4] has been employed to measure the surface roughness of the micro-heterogeneous texture in a deep hole, by virtue of the frequency domain features of microscopic images and a back-propagation artificial neural network optimized by the genetic algorithm. Indeed, neural networks, geometric, color and texture features were often employed in the classification of plants and crops [5,6,7]. Shen *et al.* [8] used the Otsu method, HSI color system and Sobel operator to extract disease spot regions and calculate leaf areas. Park *et al.* [9] employed an unsupervised segmentation algorithm by Gaussian mixture models to segment color image regions.

However, to the best of the authors’ knowledge, there is no literature on the inspection of the internal quality of micro-spray nozzles using image processing techniques. Some research [10,11,12] talked about the determination of drop sizes and shapes for spray nozzles. The drop size distribution of an irrigation spray nozzle was determined using image processing techniques, and the relationship between drop size distribution, operating pressure and nozzle size were discussed [10]. The droplet shape and size of a diesel spray had been detected using wavelet transform, a Gaussian filter and sub-pixel contour extraction [11]. The gray level gradient, boundary curvature detection, the Hough transform and the convex-hull method were employed to count and size particles in the field of sprays [12].

Currently, the internal damages of micro-spray nozzles are manually inspected (sorted) using stereomicroscopes. The procedures are laborious and ineffective. The labor cost was about 80% of the total cost for micro-spray nozzle products. The detection process has to be automated to reduce the total cost. Thus, this paper aims to develop a machine vision system to correctly and automatically inspect the micro-spray nozzle. The specific goals are: (1) to develop an algorithm to segment the defect region; (2) to extract the gray level and texture features; and (3) to classify different grades using the aforementioned features by a classifier.

In summary, we develop a machine vision system to correctly and automatically inspect and classify micro-spray nozzles. The system includes an extraction algorithm of defect regions, a feature estimation algorithm for defect regions and a classifier for the quality of micro-spray nozzles.

## 2. Materials and Methods

### 2.1. Micro-Spray Nozzle Structure and Defects

**Figure 1:**
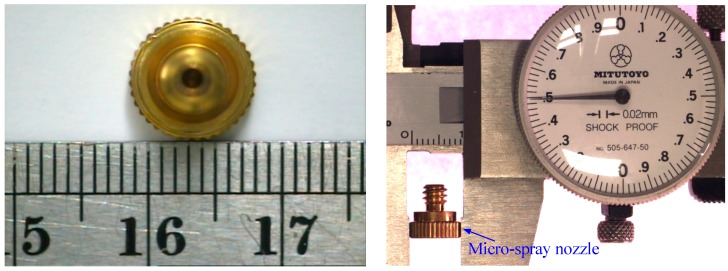
Micro-spray nozzle.

**Figure 2:**
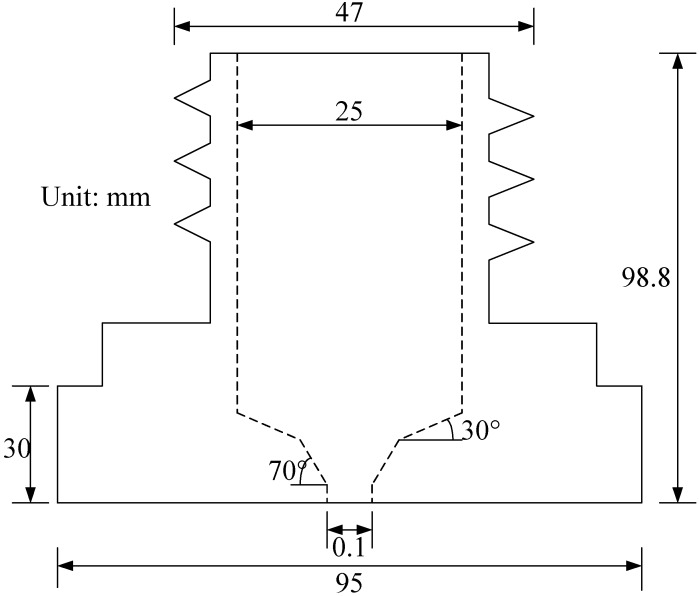
The profile of the micro-spray nozzle.

**Figure 3:**
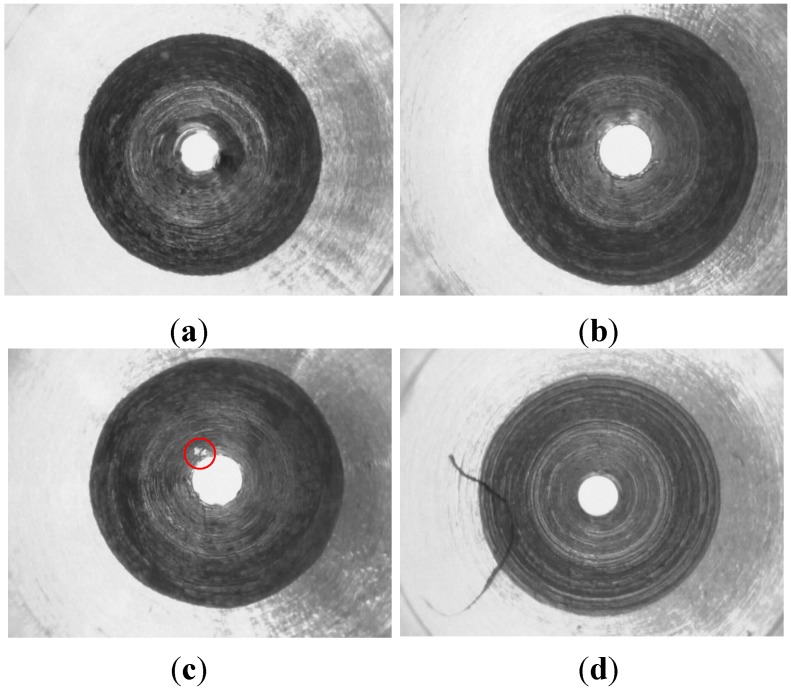
Four possible defects. (**a**) Small outlet; (**b**) Deckle edge of outlet; (**c**) Pellet metal fillings; (**d**) Strip metal fillings.

Micro-spray nozzles (shown in Figure 1) are provided by Natural Fog Multi-Tech Precision Industry Corporation Ltd. (Tai-Chung, Taiwan). The structure profile of the micro-spray nozzle is shown in Figure 2. The outlet diameter of the micro-spray nozzle is 0.1 mm. There are two inclined annular-planes, A and B (as shown in Figure 2), on the inside surface of the micro-spray nozzle. Figure 3 depicts the circle textures in the inner image of micro-spray nozzles after CNC machine manufacturing. The defects of micro-spray nozzles were made by the CNC machine during the manufacturing procedures. The defects appear on the outlet and inclined planes A and B. Four possible defects, which include the outlet shape and deckle edge, pellet and stripe metal filings on inclined planes, are shown in Figure 3. The purpose of this study is to inspect the defects of micro-spray nozzles automatically using a machine vision system.

### 2.2. Hardware System

**Figure 4:**
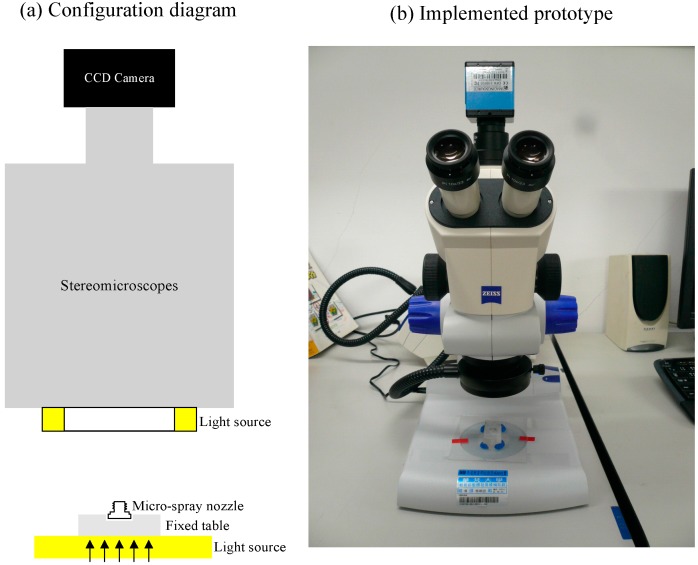
Illustration of the machine vision system for micro-spray nozzle defect inspection.

The machine vision system implemented to inspect the inner images of micro-spray nozzles is illustrated in Figure 4. This system includes an IEEE 1394 CCD color camera (DFK-31BF03, Imaging Source Inc., Bremen, Germany), a stereomicroscope (Stemi 2000-C, Zeiss Inc., Oberkochen, Germany), a front illuminating white light LED with a diffuse filter, a fixed table and a personal computer. Open Source Computer Vision Library (OpenCV1.0, Intel Corporation) and Visual C++ 6.0 programming are linked to the programs to grab images of 1024 × 768 pixels.

### 2.3. Region Segmentation and Features Extraction

Algorithms that include the estimation of the outlet’s geometric feature and segmentation of the region of interest (ROI), a circle inspection (CI) algorithm and a classifier are proposed to detect micro-spray nozzle defects. Details of the proposed algorithms are described as follows.

#### 2.3.1. Outlet Extraction

**Figure 5:**
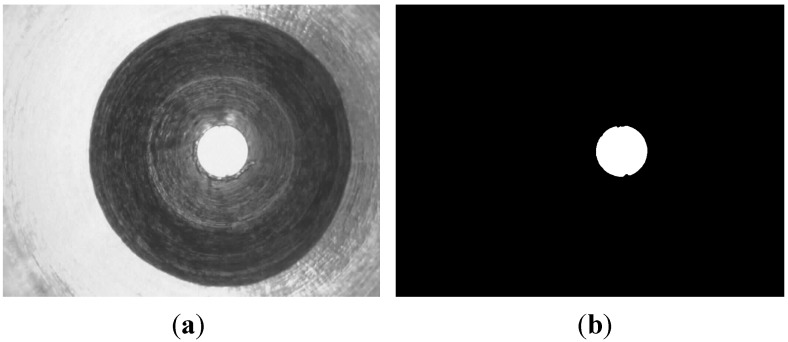
Outlet image segmentation. (**a**) Orignal image; (**b**) Outlet image.

Segmentation of the ROI image is an essential procedure once the features of the micro-spray nozzle have been extracted. Firstly, the outlet image (as shown in Figure 5) is segmented using Otsu’s auto-thresholding method and hole-filling operations [13]. By assuming that the binary image of the outlet is $f(x_{i},y_{i})$ (where *i* = 1, 2, …, *m* and the total number of pixels is *m*), the centroid is obtained as $\bar{X}=\sum_{i=1}^{m}x_{i}/m$, $\bar{Y}=\sum_{i=1}^{m}y_{i}/m$. The covariance matrix is defined as $C=(\sum_{i=1}^{m}U_{i}U_{i}^{T})/m-MM^{T}$, in which $U_{i}$ is the *i*-th coordinate vector of the image and $M=(\sum_{i=1}^{m}U_{i})/m$ is the mean vector. *T* indicates vector transposition. A pair of orthogonal eigenvectors of the covariance matrix is then calculated. The geometric features—principle axis length (*L_p_*), secondary axis (*L_s_*), centroid, area (*A*), perimeter (*P*), compactness (*P*^2^/4π*A*), diameter ratio (min./max. diameter, *D_mm_*) and mean diameter (*D_mean_*) of the outlet—are computed using eigenvectors. Secondly, two geometric features—diameter ratio and mean diameter—are employed in the micro-spray nozzle classification process.

**Figure 6:**
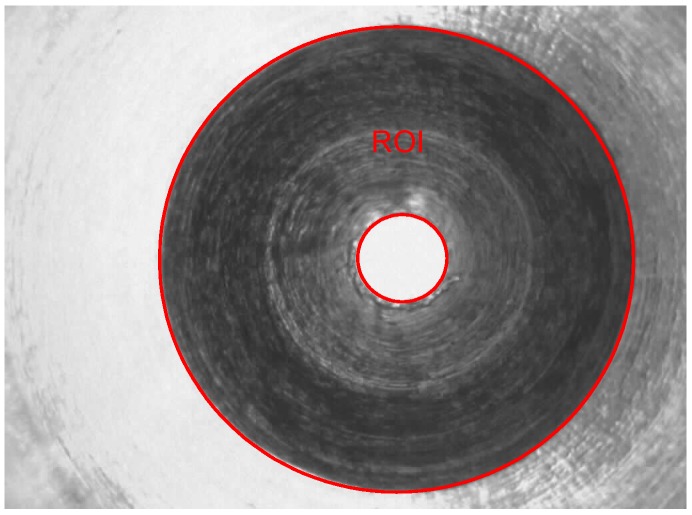
ROI diagram.

The ROI of inclined annular-planes is the internal image of the micro-spray nozzle, as shown in Figure 6. Segmenting the ROI effectively is an important procedure once the defects have been detected. Firstly, Canny edge detection [13], a randomized algorithm for detecting circles [14], hole-filling operation and the AND logic operator are used to segment the ROI of the inclined annular-planes.

The procedures of extraction are as follows.

**Figure 7:**
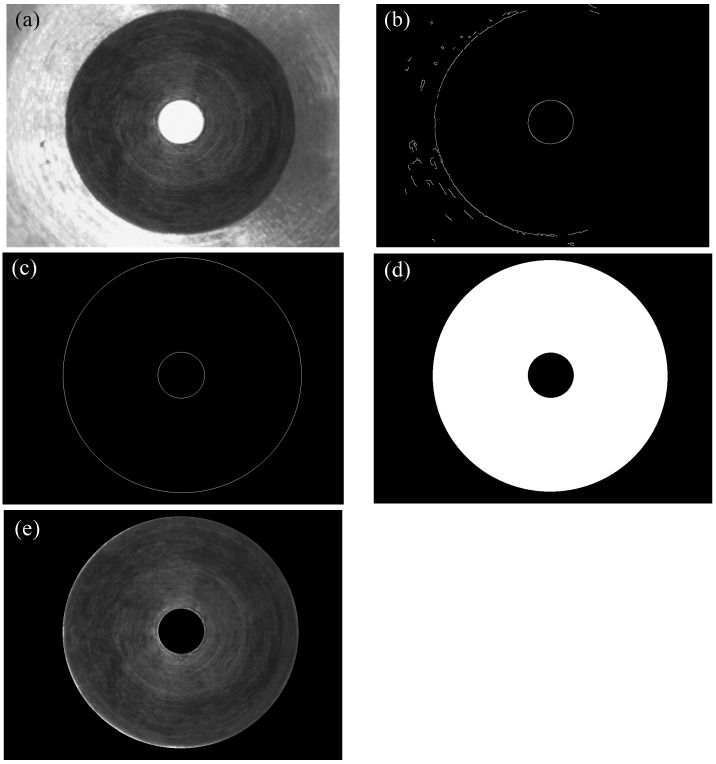
(**a**) Original image; (**b**) after Canny edge detection; (**c**) randomized algorithm for detecting circles; (**d**) the image after hole-filling operation; (**e**) ROI image with the AND logic operator for (a,d).

Step 1. Canny edge detection for the original image (Figure 7a), shown as Figure 7b.
Step 2. Randomized algorithm for detecting circles, shown as Figure 7c.
Step 3. The ring region is filled using a hole-filling operation, shown as Figure 7d.
Step 4. The ROI (shown as Figure 7e) is segmented using the AND logic operator for Figure 7a,d.
Step 5. Geometric features’ computation.

#### 2.3.2. Possible Defect Segmentation

**Figure 8:**
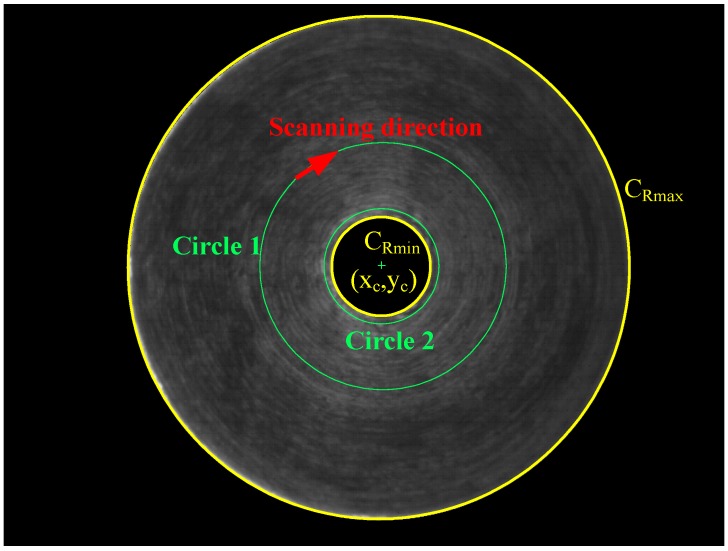
The scanning direction of the circle inspection for the ROI.

**Figure 9:**
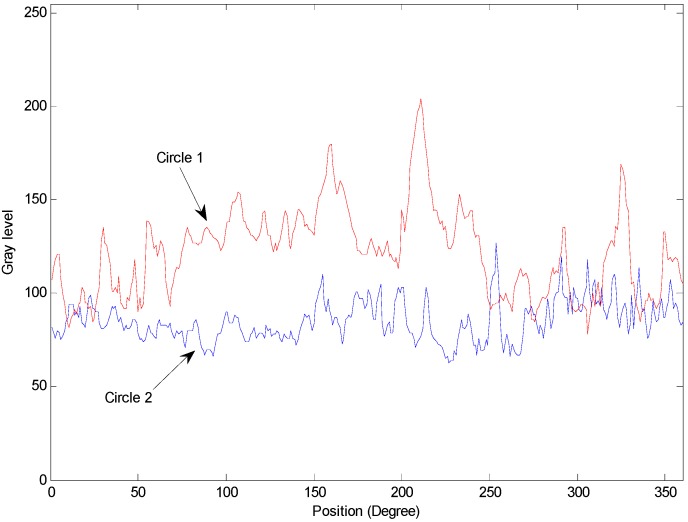
The distribution of gray levels of the circle inspection.

Segmenting defect regions is an important procedure before possible defects (including the deckle edge, pellet and stripe metal fillings) are detected and classified. A prior experiment proceeded as follows. Firstly, an inspection circle is used to find gray levels with scanning resolution in a pixel, as shown in Figure 8. There are different distribution forms of gray levels in different circles, as illustrated in Figure 9. However, the difference between defects and non-defects is difficult to distinguish using the thresholding method [13]. Therefore, a novel method, the circle inspection algorithm (CI algorithm), is proposed for defect region segmentation of micro-spray nozzles in this study.

**Figure 10:**
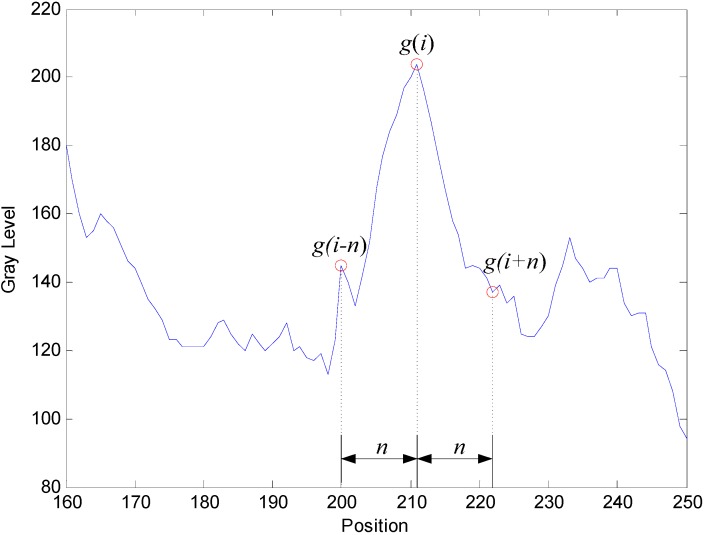
Parameters of gray level gradient computation.

The CI algorithm is effectively executed to extract defect regions for internal images of micro-spray nozzles according to the differences of the gray level gradient. The CI algorithm is described as follows:

Step 1. The distribution of the gray level on the image is estimated along the circle *C_Rj_* (with scanning resolution in a pixel); (*x_c_*, *y_c_*) is the center, and *R_min_* < *R_j_*< *R_max_*.
Step 2. Compute the gray level gradient ($G_{i}$) of the circle, as demonstrated in Figure 10 on Circle 1.
  (1) $$\begin{array}{c} G_{i}=\frac{1}{2}\left[\left(\frac{\left|g(i)-g(i-n)\right|}{n}\right)+\left(\frac{\left|g(i)-g(i+n)\right|}{n}\right)\right]\\ =\frac{1}{2n}\left|2g(i)-g(i-n)-g(i+n)\right| \end{array}$$
  where *g*(*i*) is the gray level of the circle, *i* is the position of the circle and *n* is the distance.
Step 3. Segment the possible defect regions (*PDR*).
  (2) $$\{\begin{array}{c} If\begin{array}{cc} & G_{i}\geq T,\begin{array}{cc} \begin{array}{cc} & then \end{array} & g\left(i\right)\in PDR \end{array} \end{array}\\ \begin{array}{cc} If\begin{array}{cc} & G_{i}<T, \end{array} & \begin{array}{cc} then & g\left(i\right)\in Normal \end{array} \end{array} \end{array}$$
  where *T* is a threshold value.

### 2.4. Classification: Classifier

**Figure 11:**
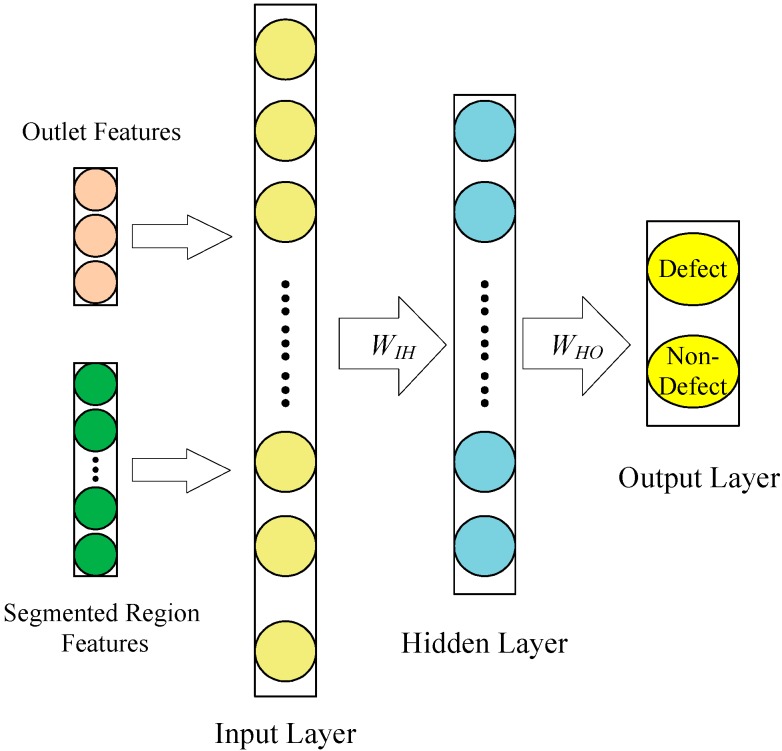
The structure of the back propagation neural network (BPNN) classifier.

Geometric, texture and color features analysis have been widely employed in classification. With proper feature selections, the design of a classifier can be greatly simplified. This study adopts the geometric feature (mean diameter, diameter ratio, distance variance), the color features (mean gray level, variance of gray level) and texture features (contrast, energy and entropy from the gray level co-occurrence matrix (GLCMs) [15]) to classify defects and non-defects using a back propagation neural network [16] (BPNN, as shown in Figure 11). Mathematical formulations of these features are given in Table 1.

**Table 1:** Mathematical formulations of the features.

Distance Variance	Variance of $\frac{1}{N}\sum_{i}^{N}\left(D(x_{i},y_{i})-\bar{D}\right)$
Mean gray level	$\bar{f}=\frac{1}{N}\overset{N}{\underset{i=1}{\sum }}f\left(x,\text{y}\right)$
Gray level variance	$\frac{1}{N}\sum_{i}^{N}\left(f(x_{i},y_{i})-\bar{f}\right)$
Contrast (orientations 45°, 90°, 135° and 180°)	$\sum_{i}\sum_{j}{\left(i-j\right)}^{2}p^{2}(i,j,d,\text{θ})$
Entropy (orientations 45°, 90°, 135° and 180°)	$-\sum_{i}\sum_{j}p(i,j,d,\text{θ})\log(p(i,j,d,\text{θ}))$
Energy (orientations 45°, 90°, 135° and 180°)	$\sum_{i}\sum_{j}p^{2}(i,j,d,\theta )$

Note: *D*(*x_i_*, *y_i_*) is the distance between location (*x_i_*, *y_i_*) and the outlet center, *p*(*i*, *j*, *d*, θ) represents the relative frequency of a gray level co-occurrence matrix (GLCM) of an image *g*(*x_i_*, *y_i_*), where *i* is the gray level at location (*x*, *y*) and *j* represents the gray level of a neighboring pixel at a distance *d* and an orientation from location (*x_i_*, *y_i_*). The GLCM of distance 1 pixel and orientations 45°, 90°, 135° and 180° are used for the isolated image.

In this study, the BPNN classifier consists of three layers: an input layer, a hidden layer and an output layer (as shown in Figure 11). The input layer has 17 nodes, which are related to 3 geometric features, 2 color features and 12 texture features, normalized between 0 and 1. The output layer is made of nodes, related to two categories: defect and non-defect. Initially, the number of nodes *n_H_* in the hidden layer is calculated using the following formula:

(3) $$n_{H}=0.5(n_{I}+n_{O})+2$$

where *n_I_* is the number of input nodes and *n_o_* is the number of output nodes. The objective of the learning process is to find a relation in a pattern that was made by the features of each segmented region. The BPNN is trained, and the weights are changed until the error convergence criterion approaches 0.1. The error signal is given by:

(4) $$E\left(t\right)=\overset{2}{\underset{k=1}{\sum }}{\left(d_{k}\left(t\right)-y_{k}\left(t\right)\right)}^{2}$$

where *d_k_*(*t*) is the desired response for neuron *k* (*k* = 1~2) and *y_k_*(*t*) is the output signal of neuron *k* at iteration *t*.

### 2.5. Overall Descriptions

**Figure 12:**
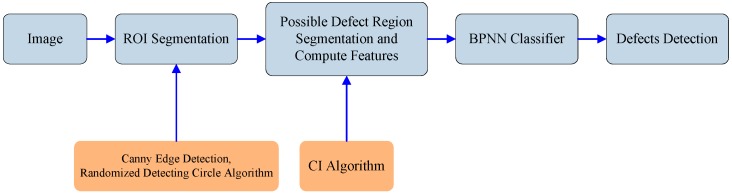
Segmentation and classification steps.

The overall steps involved in the defect region segmentation and classification are shown in Figure 12. The algorithm for the classification of defects included image processing techniques, the CI algorithm and the BPNN classifier, are fully described in the previous two sections.

The program employed for detecting and classifying the defects is written in Microsoft Visual C++ 6.0 and OpenCV 1.0.

## 3. Results and Discussion

In this paper, a defect detection (DD) software for a micro-spray nozzle is developed using image processing techniques and the CI algorithm (written in Visual C++ 6.0 with OpenCV 1.0), as outlined in the first few steps shown in Figure 12. The algorithm is summarized as follows:

Step 1. Two geometric features of the outlet are computed.
Step 2. The ROIs are segmented using Canny edge detection, a randomized algorithm for detecting circles, hole-filling and the AND logic operators.
Step 3. Possible defect regions are segmented by the CI algorithm.
Step 4. Estimate 15 features of the segmented regions.
Step 5. Establish and test the BPNN classifier to classify defects and non-defects.

The functions of DD include file operations (acquire, load and save images) and image analysis operations (binary operator, hole-filling, remove noises using closing, opening and Canny edge). The geometrics of the outlet, texture and color features of the segmented region are obtained, and defects can be detected by the DD computation accurately and rapidly.

**Figure 13:**
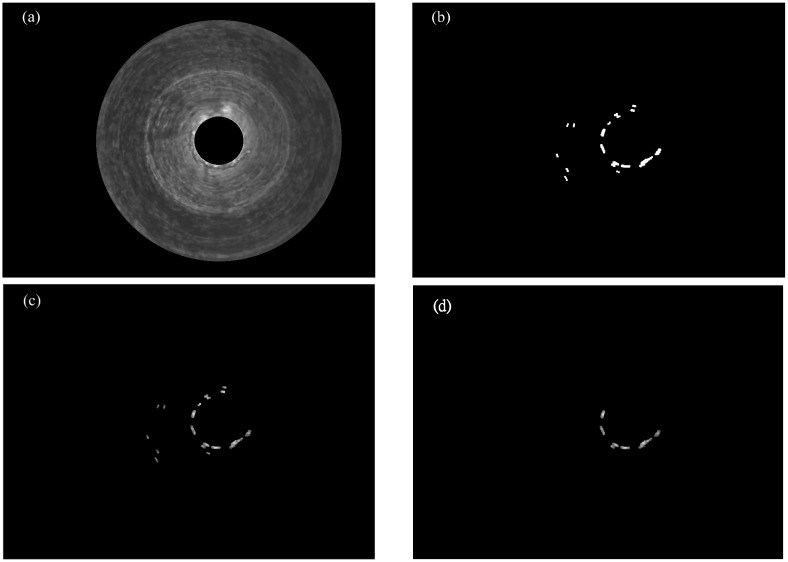
(**a**) Original image; (**b**) segmented binary image after the CI algorithm; (**c**) segmented image after the CI algorithm; (**d**) classification result.

The difference between defects and non-defect regions is not obvious in the original image of a micro-spray nozzle, as shown in Figure 13a. Possible defect regions, including defects and non-defects, are segmented using the CI algorithm (parameters *n* = 6, *T* = 11 are selected after some experimentations) and image processing techniques (such as hole-filling, erosion, dilation, opening, closing and Canny edge operators), as indicated in Figure 13b. Figure 13c results using the AND logic operator for Figure 13a,b. Hence, the defects of micro-spray nozzles (Figure 13d) are detected according to the BPNN classification.

In order to establish the BPNN classifier, 698 samples (with 565 non-defects and 133 defects) are randomly sampled. Eleven hidden nodes are obtained by Equation (3) according to 17 input features, and two output categories. The BPNN classifier is implemented using functions of MATLAB 8.0. For each configuration, the training sample set is randomly selected from all samples until the BPNN convergence is achieved. Over-fitting often occurs when the training set contains some incorrect samples in the BPNN. As the grades of micro-spray nozzles in training samples are already known before the training process, over-fitting is unlikely to occur. To ensure that the influence of over-fitting is trivial during the training process, the maximum number of iterations is set to 100,000. Further studies showed that the same inspection effect is obtained when the error rate convergence criterion is smaller than 0.1. Randomly-sampled images (1486 non-defects and 118 defects) are used to test the system. The accuracy of the classification is 90.71%. The accuracy of outlet shape and strip fillings is 100%. However, a few deckle edges and pellet fillings of the images can not be detected using the proposed algorithms, examples of classification failure and explanations are shown in Table 2.

In this study, the proposed system can detect and classify visible defects with a CCD camera accurately and efficiently. In the future, we hope that the detection and classification system can be applied to an auto-inspection system for micro-spray nozzles.

**Table 2:** Examples of classification failure and explanations.

	Original Image	ROI	Explanation
Case 1: Deckle edge	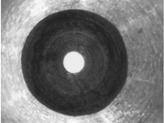	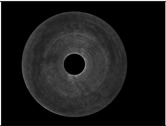	Deckle edges are removed after ROI segmentation operator.
Case 2: Deckle edge	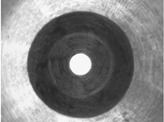	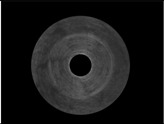	Deckle edges cannot be segmented by the CI algorithm because they are not obvious.
Case 3: Pellet filings	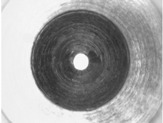	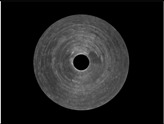	Pellet fillings cannot be segmented by the CI algorithm because they are not obvious.
Case 4: Dark image	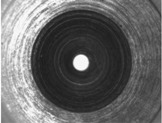	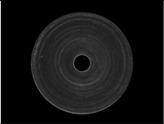	The image is blurred and dark.

## 4. Conclusions

Real-time flow uniformity depends on the internal quality of the micro-spray nozzle, and unstable flow will result because of internal defects. A novel inspection system for internal defects of micro-spray nozzles is developed in this study. The image processing techniques, Canny edge detection, a randomized algorithm for detecting circles, the circle inspection algorithm and the BPNN classifier are used to establish the micro-spray nozzle detection system. Possible defects can be segmented efficiently using the CI algorithm and image processing techniques. Geometric, color and texture features of the defects are obtained to establish a BPNN classifier. Testing results show that the defects of the micro-spray nozzles can be detected efficiently using the proposed system. In a future study, we aim to refine the inspection algorithm in order to increase the inspection accuracy for the production line of micro-spay nozzles.
